# Spinal Origin of Rheumatism

**Published:** 1833

**Authors:** 


Art. II. *Spinal origin of Rheumatism.*—We owe an apology to our readers, for having so long neglected to notice the opinions and practice of the talented Dr. John K. Mitchell, of Philadelphia, on the subject of Rheumatism. In the 8th vol. of the American Journal, he inserted a paper, intended to prove by facts and cases, that this disease originates in irritation or inflammation of the spinal marrow, particularly about the origin of the nerves leading to the affected part. Very lately, August 1833, he has favored the profession, through the same respectable periodical, with a second paper on Rheumatism, setting forth his later observations and reflections, together with those of several of his friends. Passing by his cases, which are numerous and pertinent, we shall present our readers with such an extract from the close of his communication, as will put them in possession of his ingenious pathological and therapeutic views; and enable them, as opportunities may offer, to test the reality of his discovery by their own observations. We shall be happy to be the organ of communication between them and the profession.

"In reviewing the thirty-five cases now presented to the readers of the Journal, several general truths seem worthy of particular notice. Among these not the least important is the bearing on the question of *pathological seat.* The first case, in conjunction with the cases of rheumatism produced by carious spine, reported in my first paper on this subject, leaves no doubt in my mind that a *condition of parts* exactly the same as in rheumatism, may and does exist as an effect of irritation of the great nervous masses at the centre. It remains to be proved by the opponents of this system, by equally conclusive facts, that a genuine translateable rheumatism is ever found to be independent of such a cause, or that the disease *properly called rheumatism* is ever a primary affection of the limbs or joints. The extraordinary facility with which most of the recited cases were cured by exclusive spinal treatment, goes far to establish concurrently the same truth.

For when a true inflammation of a joint has a purely local character, as in Case XXXIII. the spinal treatment is altogether useless, while the more direct application of the same means meets with prompt success. If inflammation alone of the tissues ordinarily attacked by rheumatism constituted that disease, inflammation from a sprain or blow affecting the same parts should have the same translateable character, which is so characteristic of true rheumatic inflammation, and should also be capable of passing not only to similar parts, but to the viscera, as not unfrequently happens in rheumatic cases.

"The great value of the opiate practice in rheumatism, is no small auxiliary to the arguments in favour of the central origin, because it is only by lessening nervous irritation that such a medicine can prove useful. It follows as a corollary that the best possible practice in these cases consists in spinal depletion and counter-stimulation combined with the judicious use of opiates. That such a practice has not been more generally followed in the public duty at the hospital, arose from a desire to obtain the more conclusive evidence of the truth afforded by the avoidance of obscuring complication. That being now attained, the practitioner will no longer feel justified in leaving out of his system of cure, any one of the useful auxiliaries called for by the varying contingencies of his cases.

"Another general fact is that rheumatism attacks those parts only whose nerves come out at or near to the part of the *medulla spinalis* which is in an irritated state. This was the fact in the carious cases, and in the case of Dr. Parker, of Elkton. Although all the nerves in descending along the spinal canal, must have been subjected to diseased influence, only those which *took their departure* from that part of the spine were influential in producing rheumatism, which consequently appeared at *their* sentient extremity exclusively. The same point is sustained strongly by the effect of the remedial measures, which were scarcely ever of any use when applied to remote parts of the spine, and were generally promptly beneficial, when directed to the origin of the nerves of the part inflamed.

"As far as we could ascertain, the remedial effect of the spinal treatment was most potent when applied exactly over the place of exit from the spine, of the nerves which supplied the inflamed part. It is also remarkable that the affection of the upper extremities was almost always the most easily remedied. This may be ascribed with probability to the greater effect of the cups on those parts of the spine which lie least imbedded in cellular tissue and muscular fibre.

"In conclusion, I may be permitted to advert to the close connexion between common rheumatism and certain diseases of mucous and fibrous tissues in the eyes, nose, mouth, alimentary canal, bladder, and urethra. In many cases diarrhœa and dysentery are found to alternate with rheumatism of the extremities, and particularly of the lower limbs. Wherever such cases happen, they are found to yield more readily to spinal treatment than to any other mode of cure, thus affording another proof of the spinal origin of such cases.

"On the whole it seems to me that the evidence for the pathology contended for, is much more conclusive in the present instance, than that on which the profession is accustomed to rest for much of its accredited science. As far as I can comprehend the objectors, their main difficulty lies in abandoning an unquestioned opinion, founded on prescriptive learning, and the more obvious phenomena of the disease. But even they will find it impossible to explain away the facts brought forward in favor of the new doctrine, unless they can show that in original inflammations of the periphery, the most judicious treatment consists in applications to the centre, a position fully overthrown by every abortive attempt to remedy sprains or bruises of the limbs by medicaments to the spine. Another objection lies in the absence of tenderness of such parts of the spine as are here presumed to be under irritation. If, however, that irritation transmits pain to a remote part, we ought not to expect to find it tender on pressure, but we ought to look for aggravation of the peripheral disease, an event actually produced in Dr. Parker's case. In a very few persons the spine was found tender on pressure, but these were patients who belonged to a debilitated class, in whom certain parts of the spine are almost always found too susceptible, whether they have or have not rheumatism, hysteria, or any fibrous or any uterine irritation. That tenderness so much insisted on, is but proof of an irritated condition of the *spinal braces*, the effect of weakness too highly tasked, and is totally independent of disease of the *medulla spinalis*.

"So far as this case has been discussed, the *facts* have been adduced solely on the side of the new doctrine, while its opponents have contented themselves with irrational and supposititious objections, according to a system which long since driven from other sciences, finds yet, I am sorry to say, a refuge in the temple of medical dogmatism. They admit the remarkable cures, the potent practice. They do not deny that rheumatism is produced by caries and injury of the spine, but as they do not see or feel the spinal irritation in the rest of the cases, the treatment, the relief, the victory over this obstinate malady, carry no argument to them, though sustained by analogies so striking and so profitable.

*Tabular Abstract of Cases.*

| Name. | Date of admission. | Date of cure. | Duration of treatment. | Remarks. |
|---|---|---|---|---|
| W. W. | 1st Sept. | | 1 day | |
| Eliz. Turner | 14th Sept. | | 4 days | |
| Geo. Steiner | 13th May | 18th May | 5 do. | |
| Chas. Wilson | 21st Jan. | 9th Feb. | 19 do. | Spinal treatment failed. |
| W. Barnard | 15th March | 20th Mar. | 5 do. | |
| Chas. Schrœder | 30th March | 1st April | 2 do. | |
| W. Bowes | 1st Nov. | 6th Nov. | 5 do. | |
| Anthony Della | 20th Dec. | 25th Dec. | 5 do. | |
| Christ. Rudolph | 23d Dec. | 25th Dec. | 2 do. | |
| Daniel Wilson | 30th Dec. | 2d Jan. | 3 do. | |
| John Morgan | 30th Dec. | 2d Jan. | 3 do. | |
| Charles Slater | 3d Jan. | 11th Jan. | 8 do. | |
| Andrew Franklin | 3d Dec. | 11th Feb. | 70 do. | Four relapses. |
| W. Carter | 16th Feb. | 22d Feb. | 6 do. | |
| Joseph Pratt | 4th Oct. | 6th Oct. | 2 do. | Several other relapses, discharged on 24th Nov. Total, 59 days. |
| The same | 15th Oct. | 17th Oct. | 2 do. | |
| Jos. L. Baker | 29th Nov. | 3d Dec. | 4 do. | |
| John Charles | 1st Dec. | 4th Dec. | 3 do. | |
| John Wilson | 3d Dec. | 4th Dec. | 1 do. | |
| J. M. Grezinger | 24th Oct. | 19th Dec. | 56 do. | Salivated. |
| James Carpenter | 7th Dec. | 15th Dec. | 8 do. | |
| Margaret Poston | 24th Nov. | 30th Jan. | 67 do. | Blisters and splints. |
| W. White | 23d Nov. | 27th Nov. | 4 do. | Total, 74 days; other treatment used. |
| Relapsed | 1st Dec. | 26th Dec. | 26 do. | |
| Relapsed | 2d Jan. | 16th Feb. | 34 do. | |
| John Cave | 28th Dec. | 2d Feb. | 36 do. | Splints. |
| John Henry | 2d Jan. | 22d Jan. | 20 do. | |
| W. Goldsmith | 15th Jan. | 19th Jan. | 4 do. | |
| Capt. P. | 10th Feb. | 13th Feb. | 3 do. | |
| G. W. Peirce | 13th Feb. | 15th Feb. | 2 do. | |
| M. Donnelly | 13th Feb. | 27th Feb. | 14 do. | Total, 22 days. |
| Relapsed | 2d March | 10th Mar. | 8 do. | |
| P. M'Coy | 2d Feb. | 10th Feb. | 8 do. | |
| J. Parsonage | 3d Feb. | 2d March | 7 do. | |
| W. Woodruff | 27th Jan. | 26th Feb. | 50 do. | |
| S. Summers | 25th Feb. | 3d March | 7 do. | |
| R. M'Donald | 16th Feb. | 6th Mar. | 18 do. | |

"Twenty-two of these cases were cured within eight days; and of the remaining ten cases, four were instances of frequent relapses through imprudent exposure during convalescence. At least two were supposed to complain for the purpose of remaining in the hospital, an event which not unfrequently exhibits hospital practice in a disadvantageous light. Only four cases therefore required any other than spinal treatment.

"Although cupping is the most potent mode of spinal treatment, I have, in private practice, not unfrequently found that a good rubefacient, such as a sinapism, produced great relief, and sometimes effected a cure. At the very commencement of an attack, it is often adequate to the entire removal of the pain, and consequent prevention of greater severity of symptoms."

We may add, in support of the foregoing views, that we have, at this time, a convalescent patient, who, after laboring for several days under a fever of no regular type, attended with slight cough, became singularly affected in the muscles of both arms. She was at first unable to move them without pain, and the flesh was sore on pressure. At length she became affected with a dull aching, and shortly after with constant pain, not confined to any particular part. This led us to examine the spine by pressure. The upper dorsal vertebræ were extremely tender—above and below she bore pressure without flinching. The disease was, evidently, *spinitis*. She had already been bled three times. A fourth bleeding, with the subsequent application of a blister over the affected vertebræ, rendered her convalescent. The blood was highly inflammatory.

A variety of hysterical symptoms occurred throughout the whole course of this complaint, and pròved refractory under the use of anti-spasmodics, but subsided with the fever and inflammation; thus justifying the spinal origin of hysteria, as pointed out by Dr. Taite. If the views of that gentleman and Dr. Mitchell, sustained by the case which we have cited, be correct, we are presented with an analogy between hysteria and rheumatism, which was not formerly suspected. In the first number of this Journal we have published a long, and as we think not uninteresting case of neuralgia, which originated as we supposed, in an inflammatory irritation at the origin of the trifacial nerves; a source of neuralgia first indicated, as far as we know, by Dr. Parry. Here then is another analogy, which, connected with the probable spinal origin of spasms and tetanus, would seem to connect the different maladies, which we have enumerated, into a natural group. The subject of the common origin of these various *muscular* affections, is one of deep interest, which we beg leave to commend to the attention of our brethren.

